# *DC-SIGN* gene promoter variants and IVIG treatment response in Kawasaki disease

**DOI:** 10.1186/1546-0096-11-32

**Published:** 2013-09-05

**Authors:** Michael A Portman, Howard W Wiener, Miriam Silva, Aditi Shendre, Sadeep Shrestha

**Affiliations:** 1 Department of Pediatrics, Center for Developmental Therapeutics, Seattle Children’s Research Institute, University of Washington, 1900 9th Avenue, Seattle, WA 98101, USA; 2 Department of Epidemiology, University of Alabama at Birmingham, 1665 University Boulevard, RPHB Room 217L, Birmingham 35294-0022, Alabama, USA

**Keywords:** *Kawasaki disease*, *IVIG treatment response*, *Fc*γ*R*, Coronary artery disease, DC-SIGN

## Abstract

**Background:**

Genetic variants in the inhibiting *Fc*γ*RIIB* mediate anti-inflammatory responses and influence IVIG refractoriness (IVIG-R). However, these variants are rare in Asian and Hispanic populations so other genes in the pathway could be potentially involved. IVIG is ineffective in mice lacking SIGN-R1, a related molecule to human DC-SIGN. Further, DC-SIGN is a known receptor for sialylated Fc, the component responsible for the anti-inflammatory action of IVIG. Thus, we hypothesized that DC-SIGN would also be involved in the pathway of IVIG response in Kawasaki Disease (KD) patients.

**Findings:**

A case-control approach was performed to examine the differential distribution of five single nucleotide polymorphisms (SNPs) in DC-SIGN promoter with IVIG-R among White (158 vs. 62), Asian (64 vs. 12) and Hispanic (55 vs. 20) KD patients. Distinct differences in allele frequency distributions of several variants in the DC-SIGN promoter were observed in the three ethnic groups. Further, Asians with the major allele “A” in rs2287886 were more likely (OR = 1.76, p = 0.04) to be IVIG non-responder, but this allele is a minor allele in other two ethnic groups, where the association was not apparent.

**Conclusions:**

DC-SIGN can potentially complement the role of *Fc*γ*RIIB* in the anti-inflammatory cascade involved in the IVIG response mechanism*.*

## Findings

Kawasaki Disease is a diffuse self-limited vasculitis occurring predominantly in children [1,2]. This disease can leave permanent damage to the coronary arteries, characterized by ectasia and/or aneurysm formation. Although often classified as an autoimmune disease, the precise etiology remains unknown. High dose intravenous immunoglobulin (IVIG) is the principal treatment for acute KD [3-5]. IVIG is often administered for the suppression of autoantibody-triggered inflammation in a variety of clinical settings; however, the mechanism of IVIG anti-inflammatory action in KD, as well as in other autoimmune diseases, remains elusive in humans. Elucidating the mechanisms of IVIG action is particularly important for KD, as a significant portion of patients do not respond appropriately, and are termed IVIG refractory (IVIG-R) [6-8].

In mice, human IVIG action requires sialylation at a single N-linked glycosylation site (amino acid 297) in the Fc portion of lgG as well as expression of the inhibitory Fcγ receptor 2B [9,10]. The sialylated IgG represents only 10 to 20% of human IVIG and interacts with a lectin receptor [9], specific intercellular adhesion molecule-3 grabbing nonintegrin homolog-related 1 (SIGN-R1), on inflammatory cells [11]. Similar interaction occurs with Dendritic Cell-Specific Intercellular adhesion molecule-3 Grabbing Nonintegrin (DC-SIGN), also known as CD209, the human orthologue for this receptor. DC-SIGN recognizes high-mannose glycans from a variety of pathogens, and acts as a pattern recognition receptor bridging innate and adaptive immunity. There has been some evidence demonstrating the role of DC-SIGN promoter variants in the susceptibility to or the protection against various infectious diseases, such as dengue fever, tuberculosis, and AIDS [12-14]. The IgG DC-SIGN interaction triggers a cascade, which promotes an increase in macrophage surface expression for FcγR2B [11]. This inhibitory FcγR cross-links with activating FcγRs and modifies inflammatory response [15]. Genetic studies performed in KD have implicated FcγR2B as an important component in the IVIG response [16]. However, other factors, in particular DC-SIGN, which presumably appears downstream from FcγR2B in the anti-inflammatory cascade [11], may play an important role in IVIG treatment mechanism. Previously, we have shown that the minor allele, A at IIB-120 (T/a) in inhibiting FcγR2B influence IVIG-R among whites, OR = 2.78 [1.02-7.69] [16]. Specifically, none of the IVIG-R individuals were AA homozygote but 5/124 (4%) were homozygote among responders and also the heterozygotes were more frequent (23% vs. 11%) among the white responders. However, these variants are absent in Asians and rare in Hispanic populations so other genes in the pathway could be involved in the IVIG mechanism in different populations.

Based on this hypothesis, we examined the influence of five single nucleotide polymorphisms (SNPs) in the promoter region of DC-SIGN that have been reported to a) influence the promoter activity and b) are associated with several infectious diseases [13,14,17-22], with IVIG response among KD patients of three ethnic groups - Whites, Asians and Hispanics. KD patients who were treated with IVIG and had documented response data from our ongoing cohort recruited from Seattle Children’s Hospital, Oakland Children’s Hospital, and Primary Children’s Hospital of Utah were included in this study. The demographics of the patients and cohort have been previously described in detail [15,16]. KD was diagnosed following the standard epidemiological criteria recommended by the American Heart Association and American Academy of Pediatrics (AHA/AAP) [23]. A standard treatment of IVIG (2 gm/kg) was given to all eligible patients and treatment response was determined within 11 days of initial fever. As stated in the AHA/AAP Endorsed Clinical Report, failure to respond to IVIG treatment was defined as persistent fever (temperature > 38°C) at > 36 hours from the initiation of IVIG infusion or recurrent fever at > 36 hours after completion of the initial IVIG infusion.

We genotyped five single nucleotide polymorphisms in the promoter region of DC-SIGN [14] using pyrosequencing assay in a cohort of 371 KD patients; 158 White responders and 62 non-responders, 64 Asian responders and 12 non-responders and 55 Hispanic responders and 20 non-responders recruited between 2006 and 2011. DNA samples were extracted from either saliva or blood, as previously described [16]. DNA amplification is performed with one biotinylated primer to allow for purification of a single stranded template for the pyrosequencing reaction. Following denaturation of the PCR amplicon in 0.1 M NaOH for 10 minutes, the single stranded product is immobilized to streptavidin-sepharose (Amersham Biosciences), washed and annealed with 15 pmoles of a “pyrosequencing” primer. The reaction was heated to 80C and allowed to cool to 25C. Then sequence analysis was performed by standard pyrosequencing reactions using PSQHS96A pyrosequencing instrument (Biotage, Uppsala, Sweden). The primers and probes of PCR and pyrosequencing primers listed in Table 1 were designed using Assay Design 1.0 program (Biotage, Uppsala, Sweden).

**Table 1:** **PCR and pyrosequencing primers used in the analysis of promoter variants in *DC-SIGN***

**SNP**	**Variants**^**†**^	**First round nested PCR**	**Pyrosequencing primer**
**rs#**		**(5’-3’)**	
(Nucleotide position)*			
*rs2287886*	G/a	F: Biotin AGCTTTTATTTCCCACCCTGTGATC	TAGYCTTTTGGATGACAGAT CCCTACCCA
*(-139)*		R: CTTGAAAGATCCGGCCCATCTC	
		S: GCTCTGATGCTTTCCAC	
*rs4804803*	A/g	F ATGGTCTGGGGTTGACAGGGAG	GGTRGGCAGGTAGCACCCCC AGTTCCTGG
*(-336)*		R Biotin GAGGACAGCAGCAGCTCAAAGC	
		S CCTCCACTAGGGCAAG	
*rs735240*	G/a	F Biotin CAGTAAAAGGACCCAGACAGAGTGAG	ACAYAAAATCAAATCTTACA GTGTGTACT
*(-939)*		R CCTTCAAAGGTTAGTTCTGCCTGTTC	
		S GCCTGTTCTCAAAATTC	
*rs11465360*	C/a	F Biotin CAAAGTAAAGATCAGGTCGAAAACCAC	ATGTKTGTTAAATCTCAGTT TAAAGATGGA
*(-1089)*		R GGGGTTTCTGTATGCTGGAAATG	
		S GATGTGCATTTACCAC	
*rs4804804*	*G/a*	F AGAAACCAGCAGGAACCAGCAG	TRGGCATAAGATACTCCCTC CAGTGCC
*(-1466)*		R Biotin GTCATGGCACTGGAGGGAGTATC	
		S CTGCCTCATTGCTCG	

*Nucleotide position as described in Martin et al. [14]; ^†^ second variant in small letters is the minor allele as described in HapMap Whites.

All analyses were performed for the entire set of KD patients and also separately for Whites, Asians and Hispanics, as determined by the principal component analysis (PCA) of ancestry informative markers (AIMs), as previously described [16]. Initially, for quality control, Hardy-Weinberg equilibrium (HWE) for each SNP was assessed in all KD patients and among responders and non-responders in each ethnic group. Genotype was available in more than 97% of the study participants and minor allele frequency was compared with available populations in the HapMap. A case-control analytical approach was used and chi-square test was performed to examine the differential distribution of alleles and genotypes between IVIG responders and non-responders. We also performed a stratified analysis among white KD patients (137 IVIG responders and 62 non-responders) who did not harbor the FcγR2B polymorphisms, as previously reported [16]. Further, the haplotype analyses using the 5 SNPs in the promoter region were performed in SAS/GENETICS using the PROC HAPLOTYPE procedure.

None of the SNPs deviated from HWE, even in small sample-size within ethnic sub-groups. Overall, the minor frequency of all the SNPs in our participants matched with related HapMap populations. Of the 5 known SNPs within the DC-SIGN promoter (Table 1) [14] analyzed, we identified an association of *rs2287886* with IVIG-R in Asian KD patients (Table 2), where the “A” allele showed significantly lower frequency in IVIG responders, whereas the frequency among non-responders were similar to the HapMap Asians. Of note, “A” is the major allele in Asians, but minor allele in Whites and Hispanics, where the association was not apparent. Recently, one study reported an association of rs4804803 with KD in Asians, but not with IVIG-R [24], consistent with our IVIG-R results However, the low number of subjects lacking IVIG response is unavoidable for an uncommon disease such as low-prevalent KD (incidence of 12-30 per 100,000 children in United States, of whom 15-20% are IVIG non-responders) [25].

**Table 2:** Allele and genotype distribution of five polymorphisms in the promoter of DC-SIGN among IVIG responders and non-responders in three ethnic groups

	**Minor allele frequency**			**Global test**		**Additive model**	
	HapMap*	**IVIG responders**	**IVIG non-responder**	**Allelic**	**Genotype**	**Odds ratio**	**p-value**
				**p-value**	**p-value**	**(OR)**	
All ethnic group		(n = 277)	(n = 94 )				
*rs2287886*		0.40	0.43	0.51	0.48	1.11	0.53
*rs4804803*		0.15	0.16	0.73	0.94	1.00	0.73
*rs735240*		0.42	0.38	0.36	0.09	0.92	0.37
*rs11465360*		0.06	0.06	0.98	0.98	0.91	0.96
*rs4804804*		0.35	0.37	0.56	0.61	0.90	0.58
Whites		(n = 158)	(n = 62)				
*rs2287886*	0.31	0.35	0.36	0.92	0.42	1.18	0.92
*rs4804803*	0.26	0.17	0.21	0.30	0.57	1.53	0.29
*rs735240*	0.45	0.46	0.41	0.31	0.23	0.65	0.31
*rs11465360*	0.05	0.07	0.07	0.96	0.96	0.92	0.96
*rs4804804*	0.31	0.35	0.35	0.96	0.57	0.88	0.96
Asians		(n = 64)	(n = 12)				
*rs2287886*	0.71	0.57	0.79	**0.04**	**0.08**	1.76	**0.05**
*rs4804803*	0.04	0.11	0.05	0.28	0.57	0.68	0.29
*rs735240*	0.26	0.24	0.19	0.32	0.56	0.71	0.31
*rs11465360*		0.03	0.04	0.72	0.71	1.14	0.71
*rs4804804*	0.41	0.37	0.50	0.23	0.13	1.37	0.23
Hispanics		(n = 55)	(n = 20)				
*rs2287886*	0.36	0.33	0.42	0.34	0.19	2.21	0.34
*rs4804803*		0.14	0.08	0.33	0.61	0.37	0.33
*rs735240*	0.44	0.48	0.43	0.54	0.22	0.53	0.53
*rs11465360*		0.05	0.03	0.56	0.55	0.81	0.55
*rs4804804*	0.21	0.31	0.35	0.32	0.66	0.51	0.69

*For Hispanics, the frequency from the Hapmap Mexican population is shown; OR based on the additive model for minor allele.

Polymorphisms within the promoter region of DC-SIGN have been previously associated with disease susceptibility for infections by pathogens and ethnic differences in allele frequencies have also been discussed as being genetic cause for racial differences in susceptibility to these diseases [13,17-22]. Kawasaki Disease shows substantially higher incidence rates in Asian populations, particularly Japanese and these variations can also be speculated for susceptibility. Although reported rates for IVIG refractoriness are relatively similar between Asian and cohorts of predominantly white individuals [26], our prior data suggest that genetic causes for refractoriness vary among these populations [16]. FcγR2B SNPs at -120 T/a and -386 G/c and their minor allele haplotype are associated with positive IVIG response in white KD patients. However, those particular SNPs were not detected in the Asian KD patients or their parents. In order to account for the potential confounding effect of these variants in FcγR2B polymorphisms we further performed separate analyses for white KD patients who did not harbor the variants in the two SNPs [16]. No association with DC-SIGN polymorphisms was noted in that specific subgroup. The frequency differences for the adverse *rs2287886 DC-SIGN* polymorphism in the Asian Hapmap than in White and Hispanics is interesting but should not be construed as conferring variation in IVIG refractory rates among those populations. In fact, ethnic specific refractory rates in U.S populations have not been well defined. The IVIG response in the Asian and White KD populations are both influenced by genetic alterations, although these effects could potentially occur at different genes within the pathway.

As noted, IgG binding to DC-SIGN purportedly stimulates FcγR2B transcriptional response and receptor expression [11], but other factors including polymorphisms and copy variation in the activating FcγR also influence IVIG response [15,16,27]. Polymorphisms in the activating receptor, FcγR3B, which cross links with FcγR2B, are also associated with IVIG response in these patients, although this effect appears consistent across ethnic/racial populations [15]. In particular, the FcγR3B NA1 polymorphism expressed almost exclusively on neutrophils adversely affects IVIG response. In summary, our finding with the association of rs2287886 and IVIG response in Asian KD patients potentially develops the hypothesis of the involvement of DC-SIGN. While multiple comparisons needs to be considered when testing associations with multiple SNPs, specifically in study populations of small sample size, the results from our study can be helpful in deciphering the IVIG mechanism by follow-up studies in larger KD populations or even pooling other diseases (e.g. myocarditis, Guillain-Barré Syndrome), where IVIG is still the choice of therapy.

### Ethical approval

The parent study and this genetic sub-study conformed to the procedures for informed consent (parental permission) approved by institutional review boards at all sponsoring organizations and to human-experimentation guidelines set forth by the United States Department of Health and Human Services.

## Competing interests

All the authors declare that there are no competing interests.

## Authors’ contributions

MAP and SS conceived, designed and led the study. AS assisted with the SNP genotyping and managed the genetic data. MS managed the clinical data and assisted with recruitment of patients. HWW performed the analysis and both AS and HWW assisted with the interpretation of the genetic analysis results. MAP and SS wrote the manuscript, and all authors participated in drafting the manuscript to the final version. All authors read and approved the final manuscript.
